# The genome of *Magnolia biondii* Pamp. provides insights into the evolution of Magnoliales and biosynthesis of terpenoids

**DOI:** 10.1038/s41438-021-00471-9

**Published:** 2021-03-01

**Authors:** Shanshan Dong, Min Liu, Yang Liu, Fei Chen, Ting Yang, Lu Chen, Xingtan Zhang, Xing Guo, Dongming Fang, Linzhou Li, Tian Deng, Zhangxiu Yao, Xiaoan Lang, Yiqing Gong, Ernest Wu, Yaling Wang, Yamei Shen, Xun Gong, Huan Liu, Shouzhou Zhang

**Affiliations:** 1 Laboratory of Southern Subtropical Plant Diversity, Fairy Lake Botanical Garden, Shenzhen & Chinese Academy of Sciences, Shenzhen, 518004 China; 2 State Key Laboratory of Agricultural Genomics, BGI-Shenzhen, Shenzhen, 518083 China; 3 Nanjing Forestry University, Nanjing, 210037 China; 4 Fujian Agriculture and Forestry University, Fuzhou, 350000 China; 5 University of British Columbia, Vancouver BC, Canada; 6 Xi’an Botanical Garden, Xi’an, 710061 China; 7 Zhejiang Agriculture and Forestry University, Hangzhou, 311300 China; 8 Kunming Botanical Garden, Chinese Academy of Sciences, Kunming, 650201 China; 9 Department of Biology, University of Copenhagen, DK-2100 Copenhagen, Denmark

**Keywords:** Genome, Evolution

## Abstract

*Magnolia biondii* Pamp. (Magnoliaceae, magnoliids) is a phylogenetically, economically, and medicinally important ornamental tree species widely grown and cultivated in the north-temperate regions of China. Determining the genome sequence of *M*. *biondii* would help resolve the phylogenetic uncertainty of magnoliids and improve the understanding of individual trait evolution within the *Magnolia* genus. We assembled a chromosome-level reference genome of *M. biondii* using ~67, ~175, and ~154 Gb of raw DNA sequences generated via Pacific Biosciences single-molecule real-time sequencing, 10X Genomics Chromium, and Hi-C scaffolding strategies, respectively. The final genome assembly was ~2.22 Gb, with a contig N50 value of 269.11 kb and a BUSCO complete gene percentage of 91.90%. Approximately 89.17% of the genome was organized into 19 chromosomes, resulting in a scaffold N50 of 92.86 Mb. The genome contained 47,547 protein-coding genes, accounting for 23.47% of the genome length, whereas 66.48% of the genome length consisted of repetitive elements. We confirmed a WGD event that occurred very close to the time of the split between the Magnoliales and Laurales. Functional enrichment of the *Magnolia*-specific and expanded gene families highlighted genes involved in the biosynthesis of secondary metabolites, plant–pathogen interactions, and responses to stimuli, which may improve the ecological fitness and biological adaptability of the lineage. Phylogenomic analyses revealed a sister relationship of magnoliids and Chloranthaceae, which are sister to a clade comprising monocots and eudicots. The genome sequence of *M. biondii* could lead to trait improvement, germplasm conservation, and evolutionary studies on the rapid radiation of early angiosperms.

## Introduction

The family Magnoliaceae Juss., with more than 300 species^1^ worldwide, comprises two genera, *Liriodendron* L., which includes only two species, and *Magnolia* L., which includes the others^2^. Approximately 80% of all extant Magnoliaceae species are distributed in the temperate and tropical regions of Southeast Asia, and the others are distributed in the Americas, from temperate southeast North America through Central America to Brazil^3^, forming disjunct distribution patterns^4^.

*Magnolia* is a member of the magnoliids, which constitutes one of the earliest assemblages of flowering plants (angiosperms) and occupies a pivotal position in the phylogeny of angiosperms^5^. After the early divergence of angiosperms (Amborellales, Austrobaileyales, and Nymphaeales), the rapid radiation of five lineages of mesangiosperms (magnoliids, Chloranthaceae, *Ceratophyllum*, monocots, and eudicots) occurred within a very short time frame of < 5MYA^6^, leading to unresolved/controversial phylogenetic relationships among some lineages of mesangiosperms^5^. To date, of the 323 genome sequences available for angiosperm species^7^, mostly those of plants with agronomic value, genomes are available for only five magnoliids: black pepper^8^, avocado^9^, soursop^10^, stout camphor tree^11^, and *Liriodendron chinense*^12^. Phylogenomic analyses based on these genomic data have led to controversial taxonomic placements of magnoliids. Specifically, magnoliids are resolved as sister to eudicots, with relatively strong support^11^, which is consistent with the results of a phylotranscriptomic analysis of 92 streptophytes^13^ and 20 representative angiosperms^14^. Alternatively, magnoliids are resolved as sister to eudicots and monocots, with weak support^8–10,12^, which is in agreement with the large-scale plastome phylogenomic analysis of land plants, Viridiplantae, and angiosperms^15–17^. As phylogenetic inferences rely heavily on the sampling of lineages and genes, as well as analytical methods^5^, these controversial taxonomic placements of magnoliids relative to monocots and eudicots need to be further examined with more genomic data from magnoliids.

*Magnolia* species are usually cross-pollinated with precocious pistils, resulting in a very short pollination period. Many species of this genus have relatively low rates of pollen and seed germination^18^, as well as low production of fruits and seeds, which leads to difficult regeneration of natural populations in nature^19–21^. Exacerbated by native habitat loss due to logging and agriculture, approximately 48% of all *Magnolia* species are threatened in the wild^1^. Conservation of the germplasm resources of *Magnolia* has many economic and ecological values. Most of the *Magnolia* species are excellent ornamental tree species^22^ due to their attractive flowers with sweet fragrances and erect tree shape with graceful foliage, as is the case for *M. denudata*, *M. liliiflora*, and *M. grandiflora*. *Magnolia* species also contain a rich array of terpenoids in their flowers^23^ and have considerable varieties of phenolic compounds in their bark^24^. Many *Magnolia* species, such as *M. officinalis*, *M. biondii*, *M. denudata*, and *M. sprengeri*, are cultivated for medicinal and cosmetic purposes^25^. However, the lack of a high-quality reference genome assembly for *Magnolia* hinders current conservation and utilization efforts. Genome sequences of *Magnolia* could greatly aid molecular breeding, germplasm conservation, and scientific research of the genus.

One *Magnolia* species that is cultivated for ornamental, pharmaceutical, and timber purposes is *Magnolia biondii* Pamp. (Magnoliaceae, magnoliids). *M*. *biondii* is a deciduous tree species widely grown and cultivated in the north-temperate regions of China. Its flowers are showy and fragrant, and essential oils can be extracted from them. Chemical extracts of the flower buds are used for local stimulation and anesthesia, anti-inflammatory, antimicrobial, analgesic, blood pressure-decreasing, and anti-allergic effects^25^. Modern phytochemical studies have characterized the chemical constituents of volatile oils^26^, lignin^27^, and alkaloids^28^ from different parts of *M. biondii* plants. Volatile oils contain a rich array of terpenoids, among which the main ingredients include 1,8-cineole, β-pinene, α-terpineol, and camphor^25^. These terpenoids are synthesized by terpene synthase (TPS), which belongs to the TPS gene family. In this study, we sequenced and assembled the reference genome of *M. biondii* using PacBio long read, 10X Genomics Chromium, and Hi-C scaffolding strategies. The ~2.22 Gb genome sequence of *M. biondii* represents the largest genome assembled to date for early-diverging magnoliids. This genome will support future studies on floral evolution and the biosynthesis of the primary and secondary metabolites unique to the species and will be an essential resource for understanding rapid changes that took place throughout the phylogenetic backbone of angiosperms. Finally, this information could be used to further improve genome-assisted cultivation and conservation efforts of *Magnolia*.

## Materials and methods

### Plant materials, DNA extractions, and sequencing

Fresh leaves and flower materials were collected from a 21-year-old *M. biondii* tree (a cultivated variety) at three developmental stages planted in the Xi’an Botanical Garden, Xi’an, China. The specimen (voucher number: Zhang 201801M) has been deposited in the Herbarium of Fairy Lake Botanical Garden, Shenzhen, China. Total genomic DNA was extracted from fresh young leaves of *M. biondii* using the modified cetyltrimethylammonium bromide (CTAB) method^29^. The quality and quantity of the DNA samples were evaluated using a NanoDrop™ One UV-Vis spectrophotometer (Thermo Fisher Scientific, USA) and a Qubit^®^ 3.0 Fluorometer (Invitrogen Ltd, Paisley, UK), respectively. Three different approaches were subsequently used for genomic DNA sequencing at BGI-Shenzhen (BGI Co., Ltd., Shenzhen, Guangdong, China) (Supplementary Table S1). First, high-molecular-weight genomic DNA was prepared for 10X Genomics libraries with insert sizes of 350–500 bp according to the manufacturer’s protocol (Chromium Genome Chip Kit v1, PN-120229, 10X Genomics, Pleasanton, USA). The resulting barcoded library was sequenced on a BGISEQ-500 platform to generate 150-bp reads. Duplicate reads, reads with ≥20% low-quality bases, or reads with ≥5% ambiguous (“N”) bases were filtered using SOAPnuke v. 1.5.6^30^ with the parameters “-l 10 -q 0.1 -n 0.01 -Q 2 -d –misMatch 1 –matchRatio 0.4 and -t 30,20,30,20”. Second, single-molecule real-time (SMRT) Pacific Biosciences (PacBio) libraries were constructed using the PacBio 20-kb protocol (https://www.pacb.com/) and sequenced on a PacBio RS-II instrument. Third, a Hi-C library was generated using DpnII restriction enzymes following in situ ligation protocols^31^. The DpnII-digested chromatin was end-labeled with biotin-14-dATP (Thermo Fisher Scientific, Waltham, MA, USA) and used for in situ DNA ligation. The DNA was extracted, purified, and then sheared using Covaris S2 (Covaris, Woburn, MA, USA). After A-tailing, pull down, and adapter ligation, the DNA library was sequenced on a BGISEQ-500 instrument to generate 100-bp reads.

### RNA extraction and sequencing

Young leaves (LEAF), opening flowers (FLOWER), and flower buds (BUDA and BUDB) at two developmental stages (pre-meiosis and post-meiosis) were collected from the same individual tree planted in the Xi’an Botanical Garden. Total RNA was extracted using an E.Z.N.A.^®^ Total RNA Kit I (Omega Bio-Tek), after which quality control was performed using a NanoDrop™ One UV-Vis spectrophotometer (Thermo Fisher Scientific, USA) and a Qubit^®^ 3.0 Fluorometer (Thermo Fisher Scientific, USA). All RNA samples with integrity values close to 10 were selected for cDNA library construction and next-generation sequencing. A cDNA library was prepared using a TruSeq RNA Sample Preparation Kit v2 (Illumina, San Diego, CA, USA) followed by paired-end (150 bp) sequencing on the HiSeq 2000 platform (Illumina, Inc., CA, USA) at Majorbio (Majorbio Co., Ltd., Shanghai, China). The newly generated raw sequence reads were trimmed and filtered for adapters, low-quality reads, undersized inserts, and duplicate reads using Trimmomatic v. 0.38^32^.

### Genome size estimation

We used 17 k-mer counts^33^ of high-quality reads from small-insert 10X Genomics libraries to evaluate the genome size and level of heterozygosity. First, k-mer frequency distribution analyses were performed according to the methods of Chang et al.^34^ to determine the occurrence of k-mers based on clean paired-end 10X Genomics data. GCE^35^ was then used to estimate the general characteristics of the genome, including the total genome size, repeat proportions, and level of heterozygosity (Supplementary Table S2 and Supplementary Fig. S1).

### De novo genome assembly and chromosome construction

De novo assembly was performed with five different genome assemblers: Canu v. 0.1^36^, Miniasm v. 0.3^37^, Wtdbg v. 1.1.006 (https://github.com/ruanjue/wtdbg), Flye v. 2.3.3^38^, and SMARTdenovo 1.0.0 (https://github.com/ruanjue/smartdenovo) with/without a priori Canu correction with default parameters. Based on the size of the assembled genome, the total number of assembled contigs, the N50 contig length, the maximum length of the contigs, and the completeness of the genome assembly as assessed via Benchmarking Universal Single-Copy Orthologs (BUSCO) analysis^39^ (1375 single-copy orthologs of the Embryophyta odb10 database) with a BLAST e-value cutoff of 1e–5, the genome assembly from the Miniasm assembler was selected for further polishing and scaffolding (Supplementary Table S3). The consensus sequences of the assembly were further improved using all the PacBio reads for three rounds of iterative error correction using Racon software v. 1.2.1^40^ with the default parameters, and the resultant consensus sequences were further polished using Pilon v. 1.22^41^ (parameters: –fix bases, amb –vcf –threads 32) with one round of error correction using all the clean paired-end 10X Genomics reads. The Hi-C reads were subjected to quality control measures (Supplementary Table S2) and then mapped to the contig assembly of *M. biondii* using Juicer^42^, with default parameters. A candidate chromosome-length assembly was then generated automatically using the 3D-DNA pipeline^43^ (parameters: -m haploid -s 4 -c 19 -j 15) to correct misjoins, order, and orientation and to organize the contigs from the draft chromosome assembly. Manual check and refinement of the draft assembly was carried out via Juicebox Assembly Tools^44^ (Table 1, Supplementary Fig. S2, and 10.5061/dryad.s4mw6m947).

**Table 1:** Final genome assembly based on the assembled contigs from Miniasm

	PacBio Assembly (polished)	Hi-C Assembly
Total scaffold length (Gb)		2.232
Number of scaffolds		9510
Scaffold N50 (Mb)		92.86
Scaffold N90 (Mb)		19.29
Max scaffold length (Mb)		168.50
Total contig length (Gb)	2.22	
Number of contigs	15,615	
Contig N50 (kb)	269.114	
Contig N90 (kb)	60.09	
Max contig length (kb)	2,134.98	
Complete BUSCOs	91.90%	88.50%
Complete and single-copy BUSCOs	87.00%	85.20%
Complete and duplicate BUSCOs	4.90%	3.30%
Fragmented BUSCOs	3.00%	4.40%

### Genome evaluation

The completeness of the genome assembly of *M. biondii* was evaluated by comparisons with the DNA and RNA mapping results, comparisons with the transcript unigene mapping results, and BUSCO analysis^39^. First, all the paired-end reads from the 10X Genomics and Hi-C data were mapped against the final assembly of *M. biondii* using BWA-MEM v. 0.7.10^45^. The RNA-seq reads from four different tissues were also mapped back to the genome assembly using TopHat v. 2.1.0^46^. Second, unigenes were generated from the transcript data of *M. biondii* using Bridger software^47^ with the parameters “–kmer length 25 – min kmer coverage 2” and then aligned to the scaffold assembly using the Basic Local Alignment Search Tool (BLAST)-like alignment tool BLAT^48^. Third, BUSCO analysis^39^ of the final scaffold assembly was also performed to evaluate the genome completeness of the reference genome of *M. biondii*.

### Repeat annotations

Transposable elements (TEs) were identified by a combination of homology-based and de novo approaches. Briefly, the genome assembly was aligned to the known repeat database Repbase v. 21.01^49^ using RepeatMasker v. 4.0.5^50^ and Repeat-ProteinMask^50^ at both the DNA and protein levels for homology-based TE characterization. For the de novo approach, RepeatModeler 2.0^51^ and LTR Finder v. 1.0.6^52^ were used to construct a de novo repeat library using the *M. biondii* assembly. TEs in the genome were then identified and annotated by RepeatMasker v. 4.0.5^50^, and tandem repeats were annotated using TRF v. 4.04^53^ (Supplementary Table S4, and 10.5061/dryad.s4mw6m947).

### Gene predictions

Protein-coding genes were predicted by using the MAKER-P pipeline v. 2.31^54^ based on de novo predictions, homology search results, and transcriptome evidence. For de novo gene prediction, GeneMark-ES v. 4.32^55^ was first used for self-training, with the default parameters. Second, the alternative spliced transcripts obtained by a genome-guided approach by using Trinity with the parameters “–full_cleanup –jaccard_clip –no_version_check –genome_guided_max_intron 100000 –min_contig_length 200” were mapped to the genome by using PASA v. 2.3.3 with default parameters. The complete gene models (10.5061/dryad.s4mw6m947) were then selected and used for training Augustus^56^ and SNAP^57^. The models were used to predict coding genes on the repeat-masked *M. biondii* genome. For homologous comparisons, protein sequences from *Arabidopsis thaliana*, *Oryza sativa*, *Amborella trichopoda*, and two related species (*Cinnamomum kanehirae* and *Liriodendron chinense*) were provided as protein evidence.

**Fig. 1:**
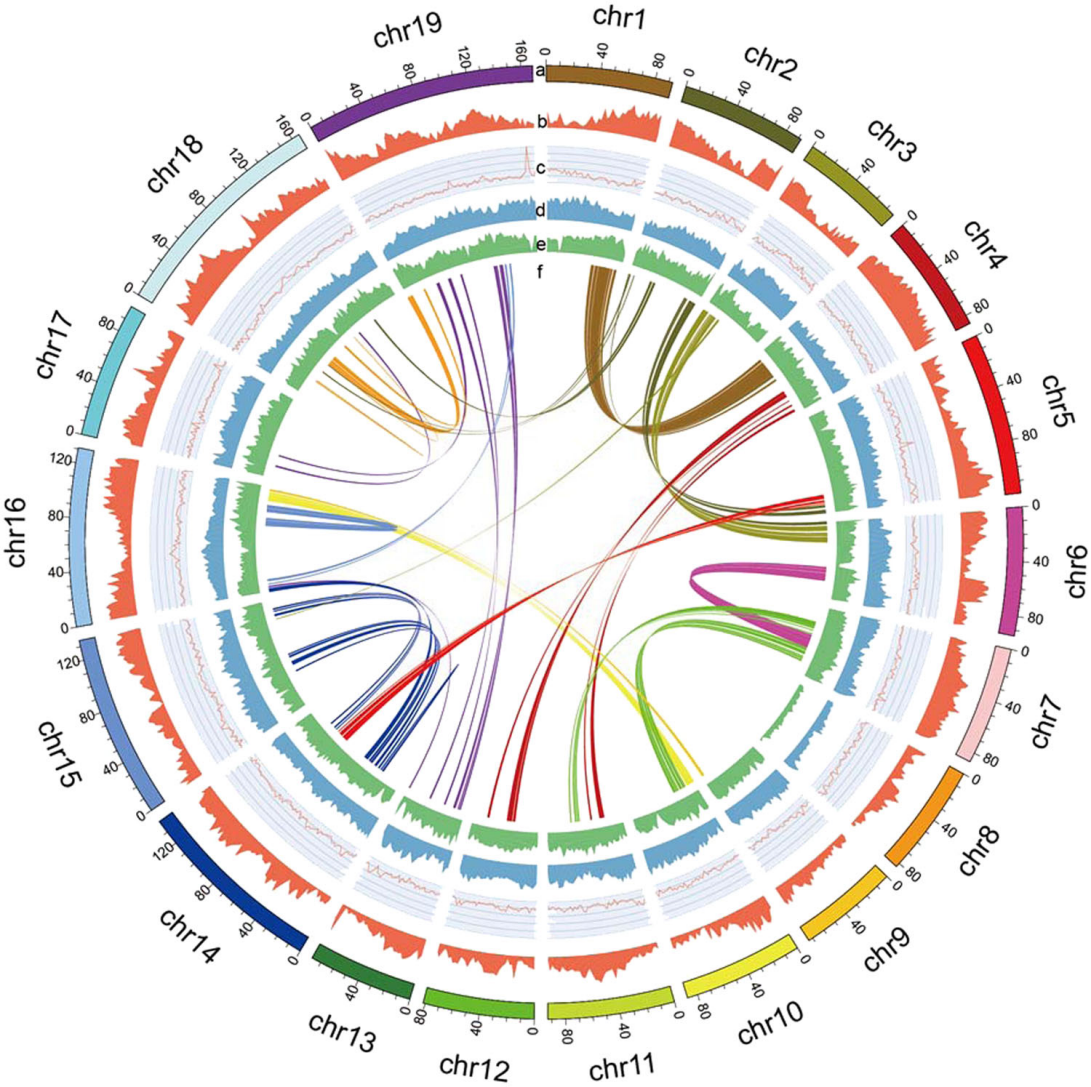
Reference genome assembly of 19 pseudochromosomes. **a** Assembled pseudochromosomes, **b** gene density, **c**. GC content, **d** *Gypsy* density, **e** *Copia* density, and **f** chromosome synteny (from outside to inside)

For RNA evidence, a completely de novo approach was chosen. The clean RNA-seq reads were assembled into inchworm contigs using Trinity v. 2.0.6^58^ with the parameters “–min_contig_length 100 –min_kmer_cov 2 –inchworm_cpu 10 –bfly_opts “-V 5 –edge-thr = 0.05 –stderr” –group_pairs_distance 200 –no_run_chrysalis” and then provided to MAKER-P as expressed sequence tags (Supplementary Fig. S3, and 10.5061/dryad.s4mw6m947). After two rounds of MAKER-P, a consensus gene set was obtained. tRNAs were identified using tRNAscan-SE v. 1.3.1^59^. snRNAs and miRNAs were detected by searching the reference sequence against the content of the Rfam database^60^ using BLAST^61^ and rRNAs were detected by alignment with BLASTN^61^ against known plant rRNA sequences^62^ (Supplementary Table S5). We also mapped the gene density, GC content, *Gypsy* density, and *Copia* density onto the individual chromosomes using the Circos tool (http://www.circos.ca) (Fig. 1).

### Functional annotation of protein-coding genes

Functional annotation of protein-coding genes was performed by searching the predicted amino acid sequences of *M. biondii* against the contents of public databases based on sequence identity and domain conservation. The sequences of protein-coding genes were previously searched within several protein sequence databases, including the Kyoto Encyclopedia of Genes and Genomes (KEGG)^63^, National Center for Biotechnology Information (NCBI) nonredundant (NR), Clusters of Orthologous Groups (COG)^64^, SwissProt^65^, and TrEMBL^65^ databases, for best matches using BLASTP with an e-value cutoff of 1e − 5. InterProScan 5.0^66^ was then used to characterize protein domains and motifs based on data acquired from Pfam^67^, SMART^68^, PANTHER^69^, PRINTS^70^, and ProDom^71^ (Supplementary Table S6).

### Gene family construction

**Fig. 2:**
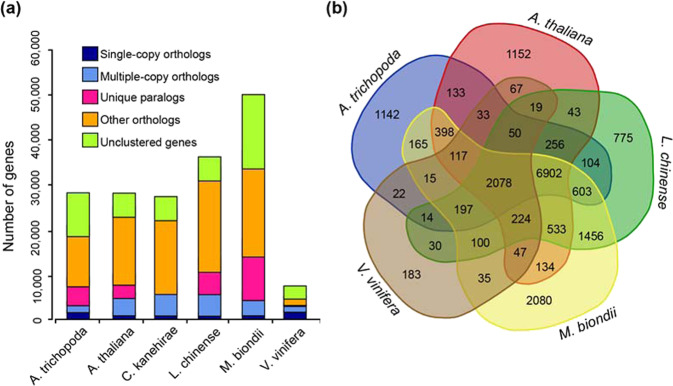
Comparative analysis of the *M. biondii* genome. **a** Number of genes in various plant species, showing a high gene number for *M. biondii* compared to a model species (*Arabidopsis thaliana*) and other species (*Amborella trichopoda*, *Cinnamomum kanehirae*, *Liriodendron chinense*, and *Vitis vinifera*). **b** Venn diagram showing overlaps of gene families between *M. biondii*, *L. chinense*, *A. trichopoda*, *A. thaliana*, and *V. vinifera*

Protein and nucleotide sequences from *M. biondii* and five other angiosperms (*A. trichopoda*, *A. thaliana*, *C. kanehirae*, *L. chinense*, *Vitis vinifera*) were used to construct gene families using OrthoFinder^72^ (https://github.com/davidemms/OrthoFinder) based on an all-versus-all BLASTP alignment with an e-value cutoff of 1e − 5. Potential gene pathways were obtained via gene mapping against the KEGG database, and Gene Ontology (GO) terms were extracted from the corresponding InterProScan or Pfam results (Fig. 2).

### Phylogenomic reconstruction and gene family evolution

**Fig. 3:**
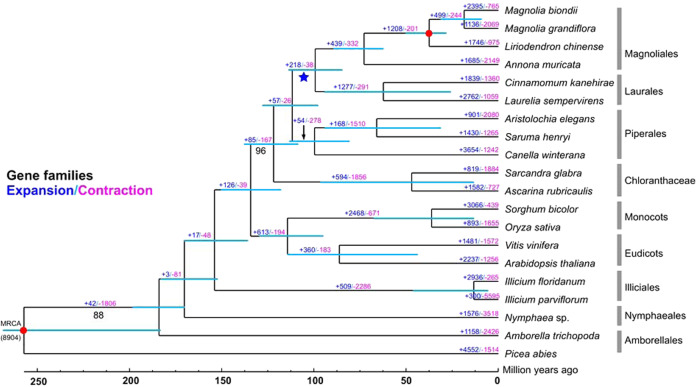
Phylogenetic tree and number of gene families that have expanded or contracted among 20 plant species. Estimated divergence time confidence intervals are shown at each internal node as teal bars. Calibrated nodes are indicated by red dots. The WGD shared between the Magnoliales and Laurales is indicated with a blue star. All the branches are maximally supported by maximum likelihood analysis unless otherwise indicated below the branches

To understand the relationships between the *M. biondii* gene families and those of other plant species and the phylogenetic placements of magnoliids among angiosperms, we performed a phylogenetic comparison of genes among different species along a 20-seed plant phylogeny reconstructed with a concatenated amino acid dataset derived from 109 single-copy nuclear genes. Putative orthologous genes were constructed from 18 angiosperms (two eudicots, two monocots, two Chloranthaceae species, eight magnoliid species, two *Illicium* species, *A. trichopoda*, *Nymphaea* sp.) and the gymnosperm outgroup *Picea abies* (Supplementary Table S7) using OrthoFinder^72^ and compared with protein-coding genes from the genome assembly of *M. biondii*. The total one-to-one orthologous gene sets were identified and extracted for alignment using MAFFT v. 5.0^73^, further trimmed using GBlocks 0.91b^74^, and concatenated by Geneious 10.0.2 (www.geneious.com). The concatenated amino acid dataset from 109 single-copy nuclear genes (each with >85% taxon occurrences) was analyzed using PartitionFinder^75^, with an initial partitioning strategy for each gene for the optimal data partitioning scheme and associated substitution models, resulting in 18 partitions. The concatenated amino acid dataset was then analyzed using the maximum likelihood (ML) method with RAxML-VI-HPC v. 2.2.0^76^ to determine the most reasonable tree. Nonparametric bootstrap analyses were performed by PROTGAMMAAUTO approximation for 500 pseudoreplicates (Fig. 3).

The best ML tree was used as a starting tree to estimate species divergence time using MCMC Tree, implemented in PAML v. 4^77^. Two node calibrations were defined by the TimeTree web service (http://www.timetree.org/), including the split between *Liriodendron* and *Magnolia* (34–77 MYA) and the split between angiosperms and gymnosperms (168–194 MYA). The orthologous gene clusters inferred from the OrthoFinder^72^ analysis and phylogenetic tree topology constructed using RAxML-VI-HPC v. 2.2.0^76^ were inputted into CAFE v. 4.2^78^ to determine whether significant expansion or contraction occurred in each gene family across species.

### Analyses of genome synteny and whole-genome duplication (WGD)

**Fig. 4:**
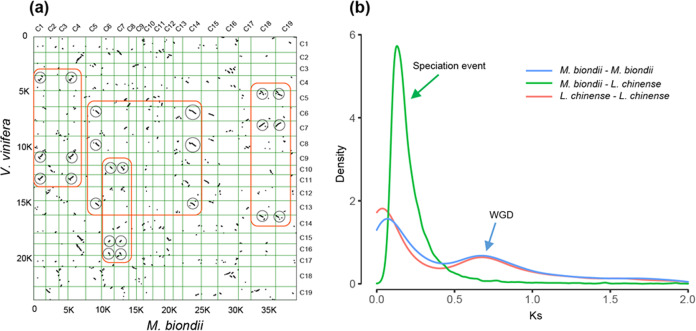
Evidence for WGD events in *M. biondii*. **a** Comparison of *M. biondii* and grape genomes. Dot plots of orthologs showing a 2–3 chromosomal relationship between the *M. biondii* genome and grape genome. **b** Synonymous substitution rate (Ks) distributions of paralogs and orthologs retrieved from gene family clustering results from OrthoMCL for *M. biondii* and *Liriodendron chinense*

To investigate the source of the large number of predicted protein-coding genes (47,547) in *M. biondii*, WGD events were analyzed by making use of the genome sequences of *M. biondii*. Given that the grape genome has one well-established whole-genome triplication and the cofamilial *L. chinense* has one reported WGD event^12^, the protein-coding genes (of CDSs and their translated protein sequences) of *M. biondii*, *L. chinense*, and grape were used to perform synteny searches with MCscanX^79^ (python version), with at least five gene pairs required per syntenic block. The resultant dot plots were examined to predict the paleoploidy level of *M. biondii* compared with that of the other angiosperms by determining the syntenic depth in each genomic region (Supplementary Figs. S4 and S5). For synonymous substitution rate (Ks) distribution analysis, the gene family clustering results of OrthoMCL^80,81^ were sorted, and gene families with only one member of both *L. chinense* and *M. biondii* and gene families with two members of either species were extracted (Supplementary Fig. S4). PAML^77^ software was then used to calculate the Ks values for the gene pairs (Fig. 4b).

### Identification of TPS genes and expression analysis

**Fig. 5:**
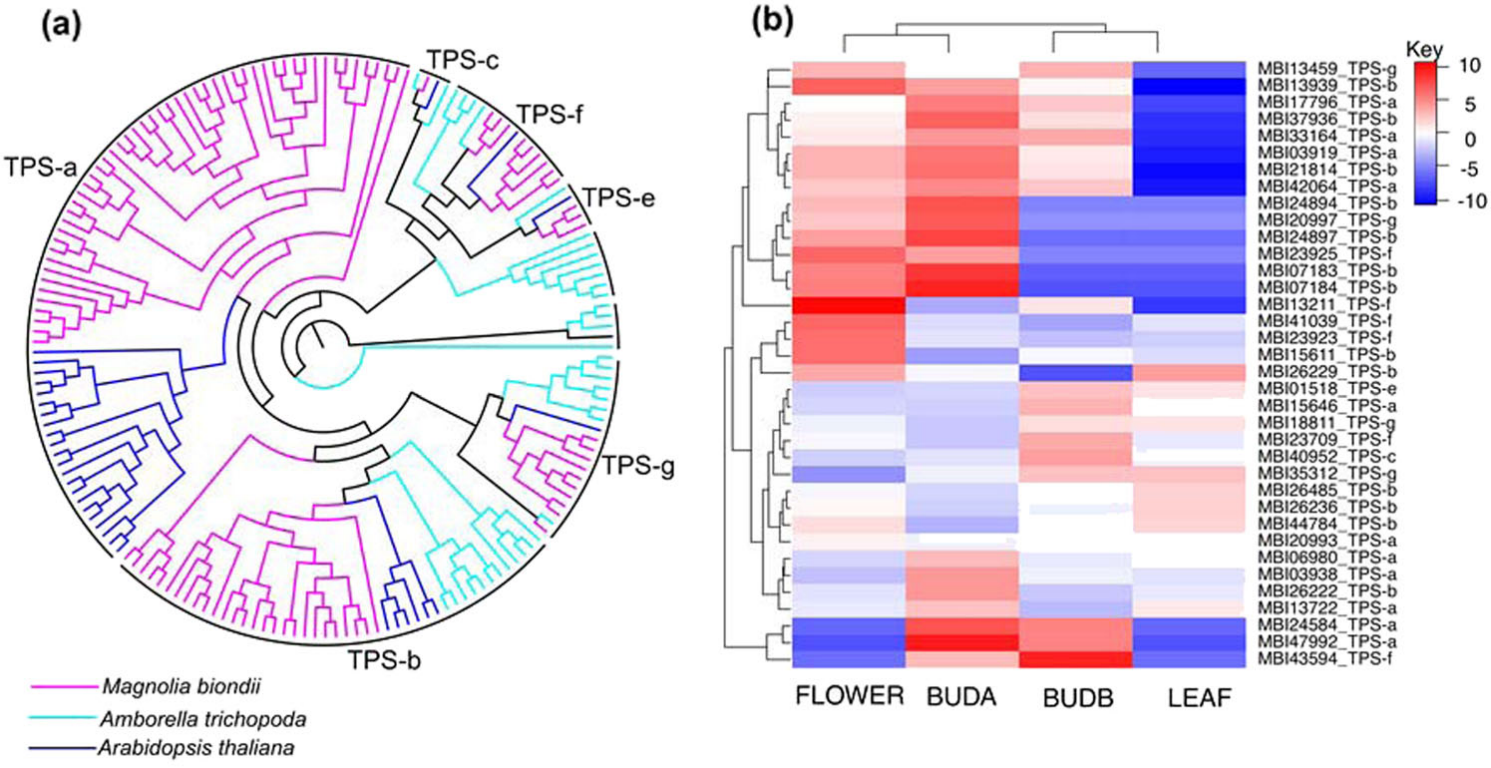
TPS gene family in *M. biondii*. **a** Phylogenetic tree of TPS genes from *Amborella trichopoda* (38 genes), *Arabidopsis thaliana* (32 genes), and *M. biondii* (102 genes). **b** Heatmap showing the differential expression of TPS genes according to the transcriptome data from young leaves (LEAF), opening flowers (FLOWER), flower buds pre-meiosis (BUDA), and flower buds post-meiosis (BUDB)

We selected two species (*A. trichopoda* and *A. thaliana*) to perform a comparative TPS gene family analysis together with *M. biondii*. Previously annotated TPS genes of the two species were retrieved from the data deposited by Chaw et al.^11^. Two Pfam domains, PF03936 and PF01397, were used as queries for searching against the *M. biondii* proteome (of the contig version) using HMMER v. 3.0, with an e-value cutoff of 1e−5^82^. The putative protein sequences of 102 TPS genes were aligned using MAFFT v. 5^73^ and manually adjusted using MEGA v. 4^83^. A phylogenetic tree was constructed using IQ-TREE^84^ with 1000 bootstrap replicates (Fig. 5).

Bowtie2^85^ was used to map the RNA-seq reads to the protein-coding sequences of the gene set. eXpress^86^ software was used to calculate the expression results of different tissues, and edgeR^87^ was used for analysis of differentially expressed genes. Parameter thresholds including an FDR of <0.001 and a log_2_FC > 2 or a log_2_FC < −2 were applied to identify differentially expressed TPS genes in *M. biondii* (10.5061/dryad.s4mw6m947).

### Data access

The genome assembly, annotations, and other supporting data are available in the Dryad database under the 10.5061/dryad.s4mw6m947. The raw sequence data have been deposited in the China National GeneBank DataBase (CNGBdb) under Accession No. CNP0000884.

## Results

### Sequencing summary

DNA sequencing generated 33-fold PacBio single-molecule long reads (a total of 66.78 Gb, with an average length of 10.32 kb), 80-fold 10X Genomics paired-end short reads (175.45 Gb), and Hi-C data (~153.78 Gb). Transcriptome sequencing generated 4.62, 4.60, 4.67, and 4.73 Gb of raw data for young leaves, opening flowers, and flower buds at two developmental stages (pre-meiosis and post-meiosis), respectively (Supplementary Table S1).

### Determination of genome size and heterozygosity

K-mer frequency distribution analyses suggested a k-mer peak with a depth of 48 and an estimated genome size of 2.17 Gb (Supplementary Fig. S1a and Table S2). GCE^35^ analysis resulted in a k-mer peak with a depth of 29 and a calculated genome size of 2.24 Gb, an estimated heterozygosity of 0.73%, and a repetitive content of 61.83% (Supplementary Fig. S1b and Table S2). The estimated genome size of *M. biondii* is the largest among all the sequenced genomes of magnoliids.

### Genome assembly and quality assessment

The selected primary assembly from Miniasm v. 0.3^37^ had a genome size of 2.20 Gb across 15,713 contigs, with a contig N50 of 267.11 kb. After three rounds of error correction with PacBio long reads and one round of correction with 10X Genomics reads, we arrived at a draft contig assembly size of 2.22 Gb spanning 15,615 contigs, with a contig N50 of 269.11 kb (Table 1 and 10.5061/dryad.s4mw6m947). Approximately 89.17% of the contig bases were organized onto the 19 chromosomes (1.98 Gb) with ambiguous Ns representing 7,365,981 bp (accounting for 0.33% of the genome length). About 9455 contigs (0.24 Gb) were not placed (Supplementary Fig. S2). The raw scaffold assembly was further improved with PacBio long reads and 10X Genomics short reads, resulting in an assembled genome size of 2.23 Gb represented by 9510 scaffolds, with a scaffold N50 of 92.86 Mb (Table 1). Our assembled genome size of *M. biondii* is very similar to the genome size estimated according to the k-mer analysis (Supplementary Table S2).

For genome quality assessment, first, all the paired-end reads from 10X Genomics and Hi-C were mapped against the final assembly of *M. biondii*, resulting in 98.40% and 92.50% of the total mapped reads, respectively. Sequencing coverage of the 10X Genomics reads and Hi-C reads showed that more than 98.04% and 86.00% of the genome bases had a sequencing depth >10X, respectively. The RNA-seq reads from four different tissues were also mapped back to the genome assembly using TopHat v. 2.1.0^46^, resulting in 93.3%, 94.4%, 92.9%, and 93.7% of the total mapped RNA-seq reads for leaves, opening flowers, and flower buds pre-meiosis and post-meiosis, respectively. Second, unigenes generated from the transcriptomic data of *M. biondii* were aligned to the scaffold assembly. The results indicated that the assemblies covered approximately 86.88% of the expressed unigenes. Third, BUSCO analysis^39^ of the final scaffold assembly showed that 88.50% (85.20% complete and single-copy genes and 3.30% complete and duplicated genes) and 4.40% of the expected 1375 conserved embryophytic genes were identified as complete genes and fragmented genes, respectively. The results of the DNA/RNA read and transcriptome unigene mapping studies and BUSCO analysis suggested that the completeness of the reference genome of *M. biondii* was acceptable.

### Repeat annotations

We identified 1,478,819,185 bp (66.48% of the genome length) bases of repetitive sequences in the genome assemblies of *M. biondii*. LTR elements constituted the predominant repeat type, accounting for 58.06% of the genome length (Supplementary Table S4). With respect to the two LTR superfamily elements, *Copia* and *Gypsy* elements constituted 659,463,750 and 727,531,048 bp, corresponding to 45.26% and 50.66% of the total LTR repeat length, respectively. The density of *Gypsy* elements decreased with increasing density of genes, whereas the *Copia* elements were distributed more evenly across the genome and showed no obvious patterns or relationships with the distribution of genes (Fig. 1). DNA transposons, satellites, simple repeats, and other repeats constituted 130,503,028, 5,540,573, 17,626,796, and 7,240,517 bp accounting for 5.86%, 0.24%, 0.79%, and 0.32%, respectively, of the genome length.

### Gene annotation and functional annotation

The assembled genome of *M. biondii* contained 47,547 protein-coding genes, 109 miRNAs, 904 tRNAs, 1918 rRNAs, and 7426 snRNAs (Supplementary Table S5). The protein-coding genes in *M. biondii* had an average gene length of 10,980 bp, an average coding DNA sequence (CDS) length of 957 bp, and an average exon number per gene of 4.4. The various gene structure parameters were compared to those of the five selected angiosperm species: *A. trichopoda*, *A. thaliana*, *C. kanehirae*, *L. chinense*, and *Oryza sativa*. *M. biondii* had the highest predicted gene numbers and the largest average intron length (~2774 bp) among these species (Supplementary Table S5), which appears to be in agreement with the relatively large genome size of *M. biondii*. However, the relatively small median gene length (3701 bp) and intron length (525 bp) in *M. biondii* suggested that some genes with exceptionally long introns significantly increased the average gene length.

Functional annotation of protein-coding genes assigned potential functions to 39,111 protein-coding genes out of the total of 47,547 genes in the *M. biondii* genome (82.26%) (Supplementary Table S6). Among ~17.74% of the predicted genes without predicted functional annotations, some may stem from errors in the genome assembly and annotations, while others might be potential candidates for novel functions.

### Gene family construction

Among a total of 15,089 gene families identified in the genome of *M. biondii*, 10,280 genes and 1928 gene families were found to be specific to *M. biondii* (Fig. 2a). The Venn diagram in Fig. 2b shows that 2078 gene families were shared among the five species *M. biondii*, *L. chinense*, *A. trichopoda*, *A. thaliana*, and *V. vinifera*.

A KEGG pathway analysis of the *M. biondii*-specific gene families revealed marked enrichment in genes involved in nucleotide metabolism, plant–pathogen interactions, and the biosynthesis of alkaloids, ubiquinone, terpenoid-quinones, phenylpropanoids, and other secondary metabolites (Supplementary Table S8). These results are consistent with the biological features of *M. biondii*, which has rich arrays of terpenoids, phenolics, and alkaloids. According to GO analysis, the *M. biondii*-specific gene families were enriched in binding, nucleic acid binding, organic cyclic compound binding, heterocyclic compound binding, and hydrolase activity (Supplementary Table S9). These specific genes associated with the biosynthesis of secondary metabolites and plant–pathogen interactions in the *M. biondii* genome assembly may play important roles in plant–pathogen resistance mechanisms^8^ by stimulating beneficial interactions with other organisms^11^.

### Phylogenomic reconstruction

Our phylogenetic analyses based on 109 orthologous nuclear single-copy genes and 19 angiosperms plus one gymnosperm outgroup revealed a robust topology and supported the sister relationship between magnoliids and the Chloranthaceae (BPP = 96), which together formed a sister group relationship (BPP = 100) with a clade comprising monocots and eudicots. The phylogenetic tree (Fig. 3 and Supplementary Fig. S5) indicated that the Magnoliales and Laurales orders have a close genetic relationship and that they diverged ~99 MYA (84–116 MYA). The estimated divergence of the Magnoliaceae and Annonaceae in the Magnoliales clade occurred ~73 MYA (57–92 MYA), while the split of *Liriodendron* and *Magnolia* is estimated to have occurred ~38 MYA (31–50 MYA).

### Gene family evolution

The orthologous gene clusters inferred from the OrthoFinder^72^ analysis and phylogenetic tree topology constructed using RAxML-VI-HPC v. 2.2.0^76^ were inputted into CAFE v. 4.2^78^ to investigate whether significant expansion or contraction occurred in each gene family across species (Fig. 3). Among the total 15,683 gene families detected in the *M. biondii* genome, 2395 had significantly expanded (P < 0.05), and 765 had contracted (P < 0.005). KEGG pathway analysis of these expanded gene families revealed marked enrichment in genes involved in metabolic pathways, biosynthesis of secondary metabolites, plant hormone signal transduction, ABC transporters, etc. (Supplementary Table S10). By the use of GO analysis, the *M. biondii* expanded gene families were enriched in ion binding, transferase activity, metabolic processes, cellular processes, oxidoreductase activity, localization, responses to stimuli, etc. (Supplementary Table S11). The expansion of these genes, especially those associated with biosynthesis of secondary metabolites, plant hormone signal transduction and responses to stimuli, could possibly contribute to the ecological fitness and biological adaptability of the species.

### Analyses of genome synteny and WGD

A total of 1738 colinear gene pairs on 147 colinear blocks were inferred within the *M. biondii* genome (Supplementary Fig. S6a). There were 13,674 colinear gene pairs from 393 colinear blocks detected between *M. biondii* and *L. chinense* (Supplementary Fig. S6b) and 10,042 colinear gene pairs from 928 colinear blocks detected between *M. biondii* and *V. vinifera* (Fig. 4a). Dot plots of longer syntenic blocks between *M. biondii* and *L. chinense* revealed a nearly 1:1 orthology ratio, indicating a similar evolutionary history of *M. biondii* to *L. chinense*. Like *Liriodendron*, *Magnolia* may have probably also experienced a WGD event^12^ after the most recent common ancestor (MRCA) of angiosperms. The nearly 2:3 orthology ratio between *M. biondii* and grape confirmed this WGD event in the lineage leading to *Magnolia* (Supplementary Fig. S6b).

The Ks distribution for *M. biondii* paralogs revealed a main peak at approximately 0.70 Ks (~116 Ma) units, which appears to coincide with the Ks peak of *L. chinense* in our observations (Fig. 4b), indicating that these two lineages might have experienced a shared WGD in their common ancestor or two independent WGDs at a similar time. The results of one-vs-one ortholog comparisons between *Liriodendron* and *Magnolia* suggested the divergence of the two lineages occurred at approximately 0.15 Ks (~24.80 Ma) units, which largely postdates the potential WGD peak of 0.70 Ks units observed in either species, indicating that this WGD event should be shared by at least the two genera of Magnoliaceae.

### TPS genes

Volatile oils isolated from the flower buds of *M. biondii* comprise primarily terpenoid compounds that are produced by the catalytic activity of TPS enzymes. We identified a total of 102 putative TPS genes in the genome assembly of *M. biondii*, which is comparable to that of *C. kanehirae*, with 101 genes^11^. To determine the classification of TPS proteins in *M. biondii*, we constructed a phylogenetic tree using all the TPS protein sequences from *M. biondii*, *A. thaliana*, and *A. trichopoda*. These TPS genes found in *M. biondii* could be assigned to six subfamilies: TPS-a (52 genes), TPS-b (27 genes), TPS-c (1 gene), TPS-e (3 genes), TPS-g (10 genes), and TPS-f (9 genes) (Fig. 5a). We compared the expression profiles of TPS genes in the young leaves and flowers at three different developmental stages (Fig. 5b) and identified a total of 36 TPS genes (11, 13, 1, 1, 6, and 4 genes for the subfamilies of TPS-a, TPS-b, TPS-c, TPS-e, TPS-f, and TPS-g, respectively) that were substantially expressed, among which 33 TPS genes (including 10 genes for both the TPS-a and TPS-b subfamilies) exhibited higher transcript abundance in the flowers than in the leaves (Fig. 5b), suggesting that these genes may be involved in a variety of terpenoid metabolic processes during *M. biondii* flower growth and development.

## Discussion

The genome of *M. biondii* is relatively large and complex, as k-mer frequency analysis suggested an estimated genome size of 2.24 Gb, with an estimated heterozygosity of 0.73% and a repeat content of 61.83%. Compared with an estimated heterozygosity of 0.087% for the genome of, for example, *Oropetium thomaeum*^88^, the heterozygosity of the *M. biondii* genome is approximately ten times higher, which probably contributed to the low contiguity of the assembly. Our DNA sequencing generated approximately 33-fold PacBio long-read data, which resulted in an assembly of 2.23 Gb spanning 15,615 contigs, with a contig N50 of 269.11 kb. The small contig N50 length might imply a fragmentary and incomplete genome assembly, which might affect the quality and precision of the Hi-C assembly. Indeed, when these contigs were organized into chromosomes using Hi-C data, approximately 6,899 contigs adding up to 1.00 Gb were disrupted by the Hi-C scaffolding processes, contributing to 0.18 Gb of genome sequence being discarded. After manual correction of the Hi-C map in Juicebox, the final scaffold assembly still showed 6911 contigs disrupted, 2358 genes disturbed, and 0.24 Gb of genome sequences not placed. BUSCO assessments showed decreased percentages of complete BUSCOs and increased percentages of fragmented BUSCOs for the scaffold assembly compared with the contig assembly (Table 1). Therefore, we used the Hi-C assembly for chromosome collinearity analysis and the contig assembly for the rest of the comparative analyses. The exceptionally large protein gene set predicted for the *M. biondii* genome might be attributed to gene fragmentation problems induced by poor genome assembly and a high content of TEs, as evidenced by the dramatically short average/median CDS length of *M. biondii* compared with that of the cofamilial *L. chinense* (Supplementary Table S5).

The chromosome-scale reference genome of *M. biondii* provided information on the gene content, repetitive elements, and genome structure of the DNA on the 19 chromosomes. Our genomic data offered valuable genetic resources for both molecular and applied research on *M. biondii* and paved the way for studies on the evolution and comparative genomics of *Magnolia* and related species. Phylogenomic analyses of 109 single-copy orthologs from 20 representative seed plant genomes with a good representation of magnoliids (three out of four orders) strongly support the sister relationship of magnoliids and Chloranthaceae, which together form a sister group relationship with a clade comprising monocots and eudicots. This placement is in agreement with the plastid topology^15,16^ and the results of multilocus phylogenetic studies of angiosperms^6^ but in contrast to the placement of the sister group relationship of magnoliids with eudicots revealed by the phylogenomic analysis of angiosperms (with *Cinnamomum kanehirae* as the only representative for magnoliids)^11^ and phylotranscriptomic analysis of 92 streptophytes^13^ and of 20 representative angiosperms^14^. Multiple factors underlie the robust angiosperm phylogeny recovered in our study: (a) we used less homoplasious amino acid data rather than nucleotide sequences (especially those of the 3^rd^ codon positions) that are more prone to substitution saturation; (b) we used an optimal partitioning strategy with carefully selected substitution models, which is usually neglected for large concatenated datasets in phylogenomic analyses; and (c) we included a relatively good taxon sampling that included representatives from all eight major angiosperm lineages, with the exception of the Ceratophyllales, for which no genomic resources are available. Future phylogenomic studies with improved and more balanced lineage sampling and thorough gene sampling as well as comprehensive analytical methods would provide more convincing evidence concerning the divergence order of early mesangiosperms.

The current assembly of the *M. biondii* genome improved our understanding of the timing of the WGD event in magnoliids. Our genome syntenic and Ks distribution analyses confirmed the WGD event in the Magnoliales. This WGD occurred ~116 MYA, as estimated by Chen et al.^12^ as well as by our study, which is close to the split time of the Magnoliales and Laurales, as the two lineages diverged approximately 113–128 MYA (mean, 120 MYA) according to the TimeTree web service (www.timetree.org) and 84–116 MYA (mean, ~99 MYA) according to our dating analysis. This WGD event might have occurred shortly before the split of the Magnoliales and Laurales, as was indicated in a recent study on the genome evolution of *Litsea*^89^. However, this hypothesis needs to be further examined in light of other results, such as the absence of a WGD event in Magnoliales *Annona muricata*^10^.

The major effective component of the flower buds of *M. biondii*, a medicinal plant species, is the volatile oils comprising a rich array of terpenoids, mainly sesquiterpenoids and monoterpenoids^90^. TPS genes of the TPS-a and TPS-b subfamilies are mainly responsible for the biosynthesis of sesquiterpenoids and monoterpenoids, respectively, in mesangiosperms. Gene tree topologies of three angiosperm TPS proteins and comparisons of TPS subfamily members with those of the other angiosperms^11^ revealed expansion of TPS genes in *M. biondii*, especially for members of TPS-a and TPS-b subfamilies. Expression profiles of TPS genes in different tissues revealed 33 TPS genes, primarily of the TPS-a and TPS-b subfamilies, that were substantially expressed in flowers compared to leaves. The expansion and significant expression of these TPS genes in the TPS-a and TPS-b subfamilies in *M. biondii* are in agreement with the high accumulation of sesquiterpenoids and monoterpenoids in the volatile oils extracted from the flower buds of *M. biondii*^90^.

## Conclusion

We constructed a reference genome of *M. biondii* by combining 10X Genomics Chromium, SMRT sequencing, and Hi-C scaffolding strategies. The ~2.22 Gb genome assembly of *M. biondii*, with a heterozygosity of 0.73% and a repeat ratio of 66.48%, represented the largest genome among six sequenced genomes of magnoliids. We predicted a total of 47,547 protein-coding genes from the genome assembly of *M. biondii*, 82.26% of which were functionally annotated. Phylogenomic reconstruction strongly supported the sister relationship of magnoliids and the Chloranthaceae, which together formed a sister relationship with a clade comprising monocots and eudicots. Our new genome information should enhance the understanding of the molecular basis of genetic diversity and individual traits in *Magnolia* as well as the molecular breeding and early radiation of angiosperms.

## Supplementary information

Supplementary figures

Supplementary tables
