# Supplementary material for: The genome of Magnolia biondii Pamp. provides insights into the evolution of Magnoliales and biosynthesis of terpenoids
Source: Hortic Res. 2021 Mar 1;8:38. doi: 10.1038/s41438-021-00471-9 (PMC7917104; doi:10.1038/s41438-021-00471-9)
Supplement: Supplementary file 1 — Supplementary figures [file 41438_2021_471_MOESM1_ESM.pdf]

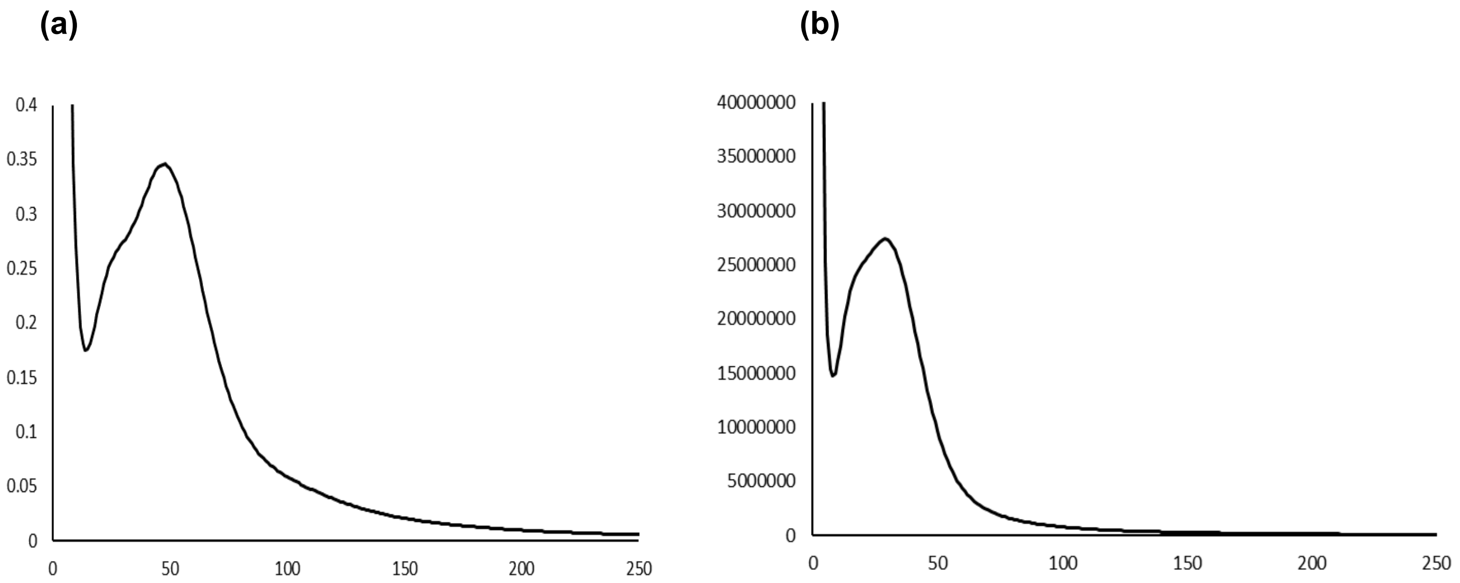

Figure S1. Estimation of the genome size of the *M. biondii* by (a) K-mer frequency analysis, and (b) GCE software

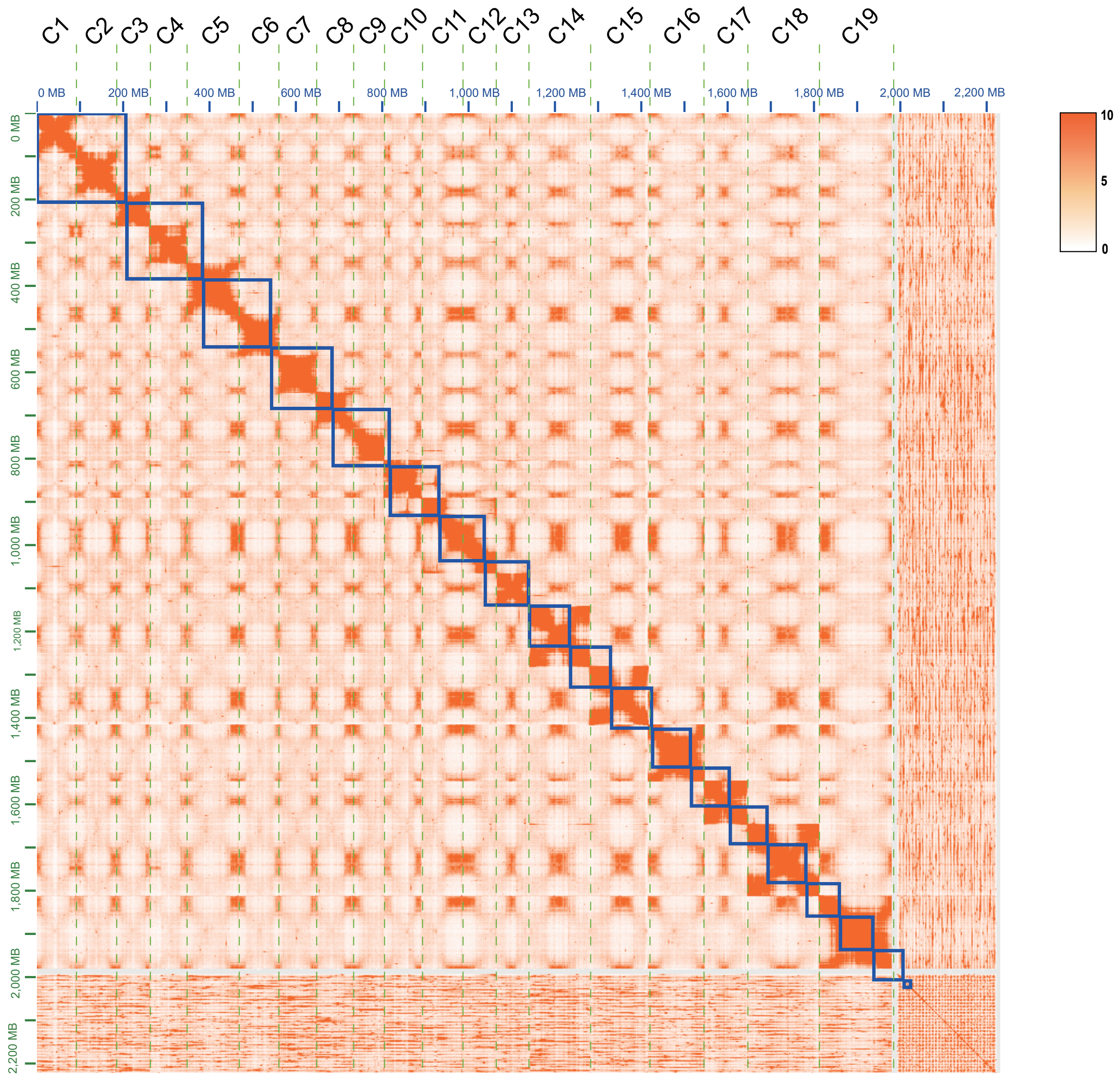

Figure S2a. Automatic Hi-C map of the *M. biondii* genome showing genome-wide all-by-all interactions. The map shows a high resolution of individual chromosomes that are scaffolded and assembled independently. The heat map colors ranging from light yellow to dark red indicate the frequency of Hi-C interaction links from low to high (0–10).

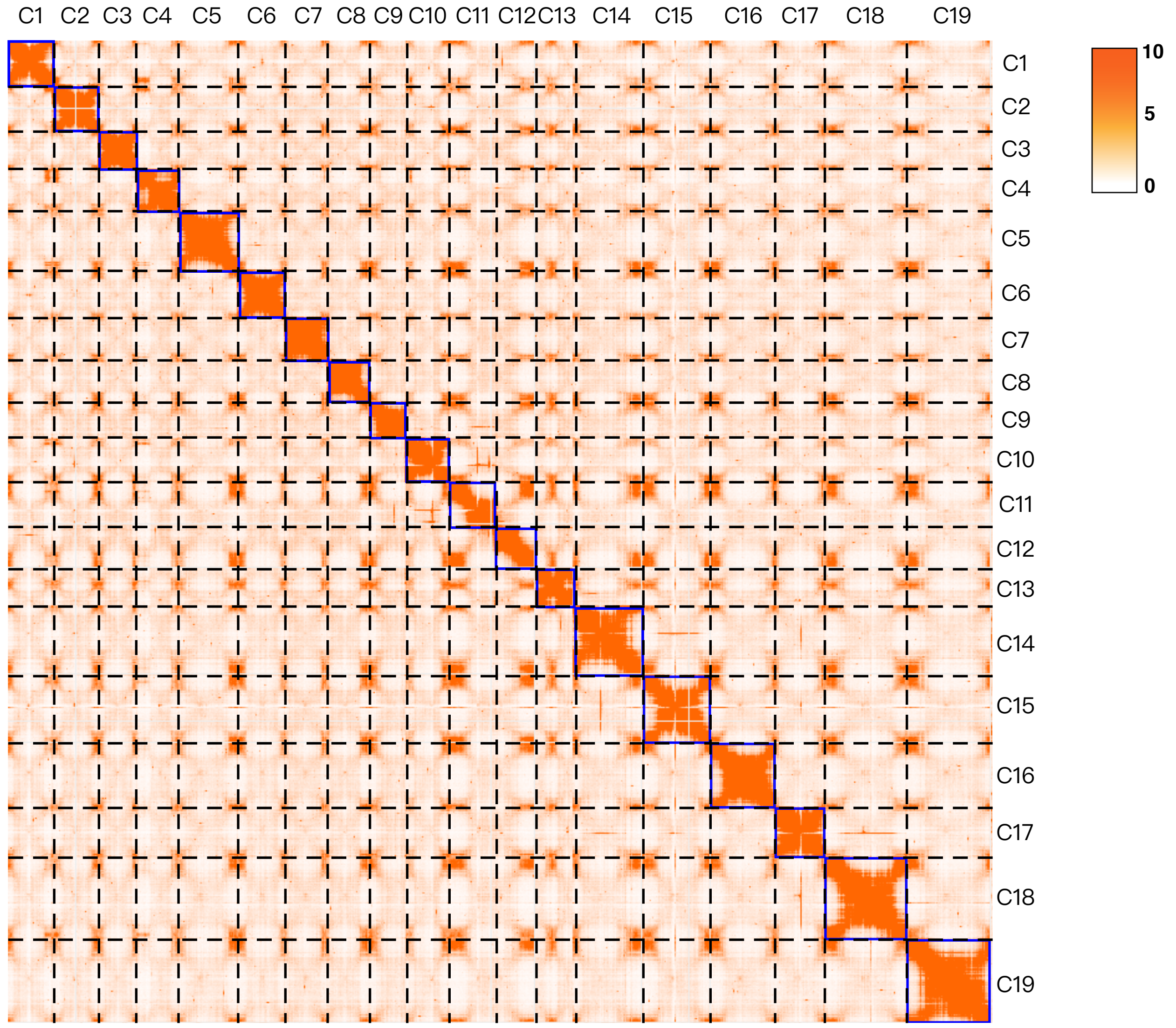

Figure S2b. Final Hi-C map of the *M. biondii* genome showing genome-wide all-by-all interactions. The map shows a high resolution of individual chromosomes that are scaffolded and assembled independently. The heat map colors ranging from light yellow to dark red indicate the frequency of Hi-C interaction links from low to high (0–10).

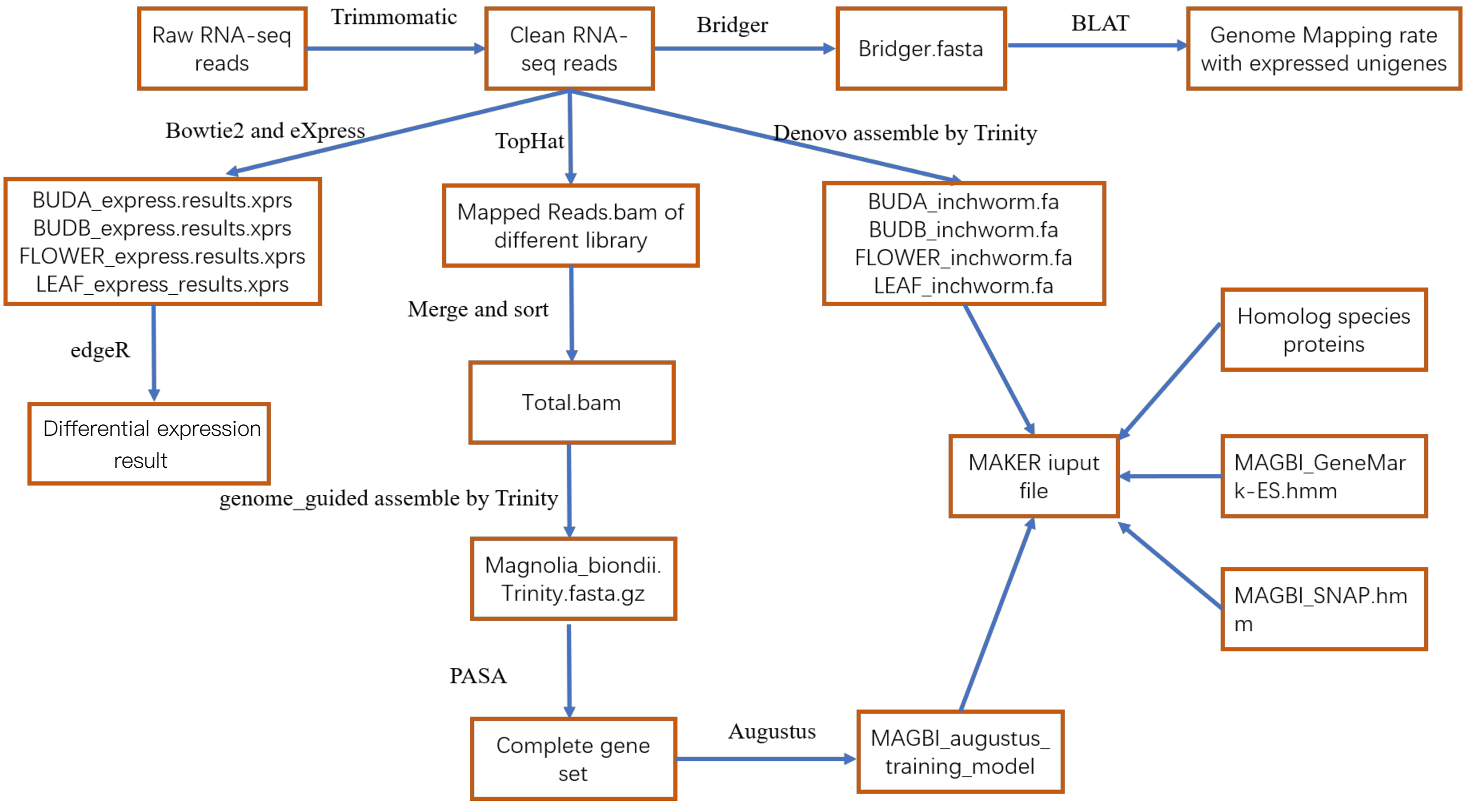

Figure S3. The data flow diagram displaying the analyses of RNA-seq data for gene prediction and differential expression of TPS genes in the floral tissues of three developmental stages. All the files mentioned above are deposited in the dryad database, and the relavant terms are defined in the README file in the data directory.

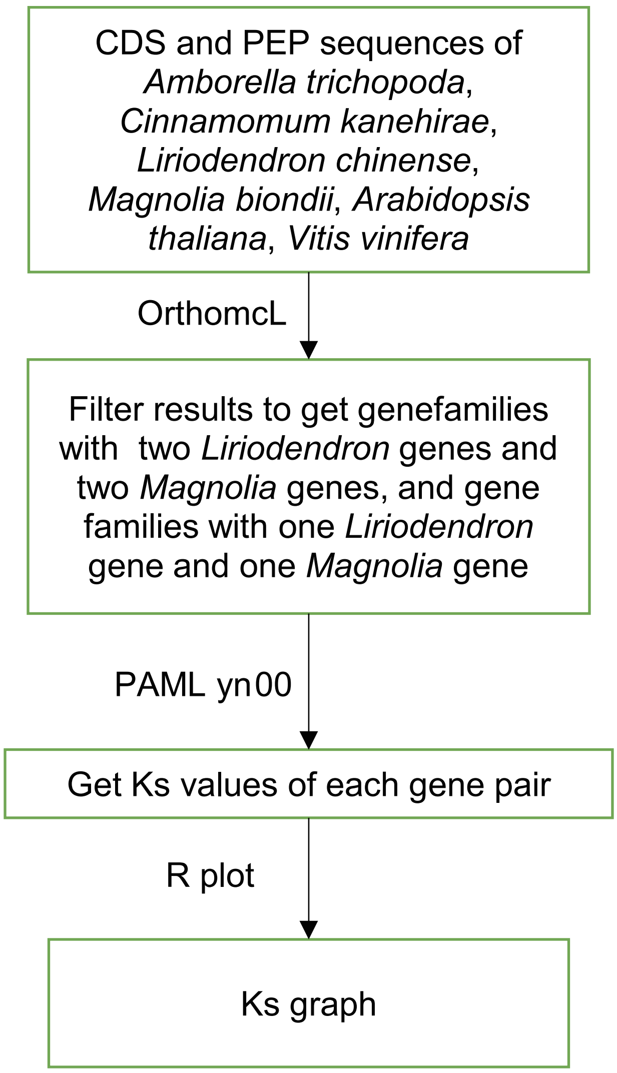

Figure S4. The flow chart displayaing the analysis of WGD.

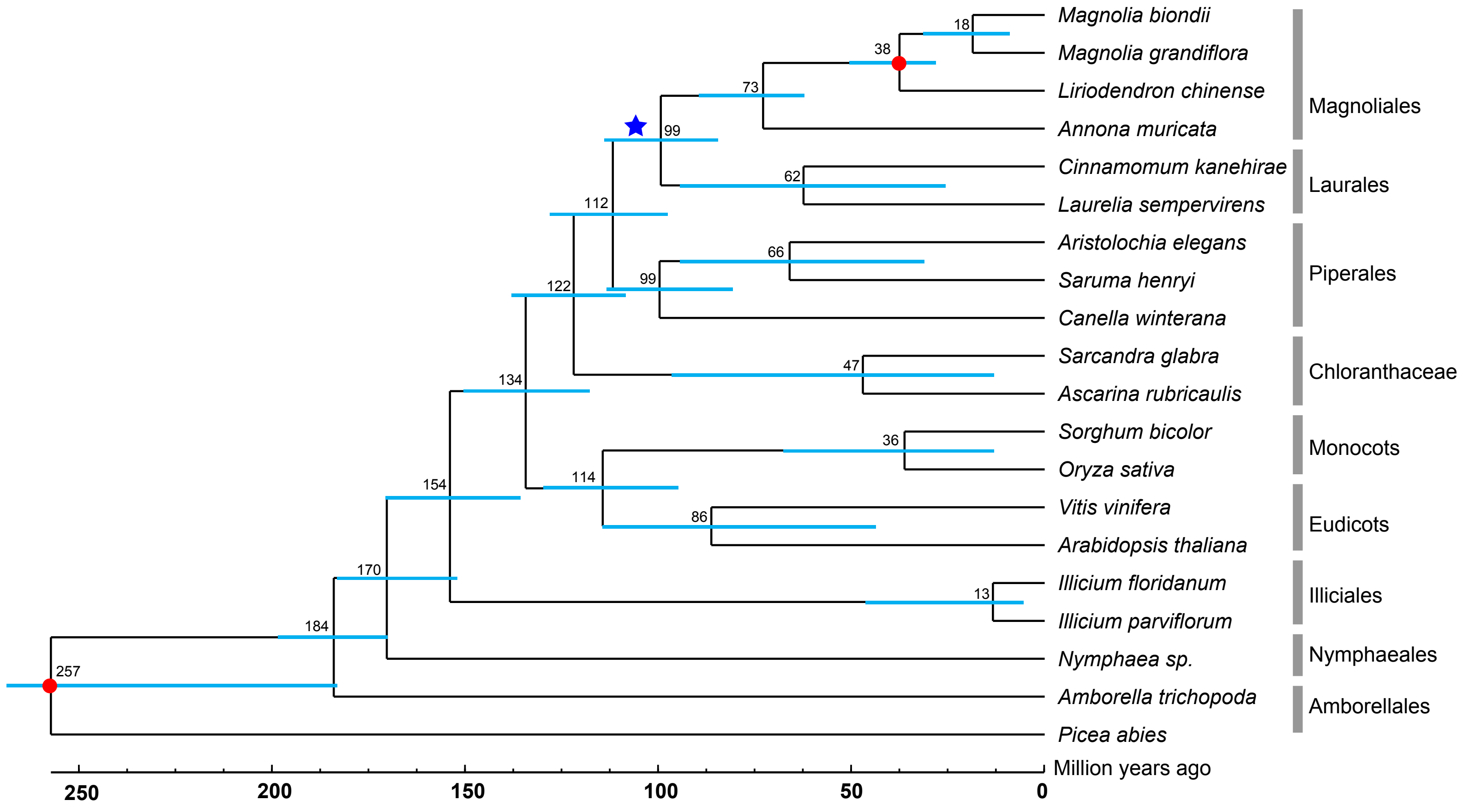

Figure S5. Divergence time estimates of magnoliids based on the ML phylogeny of 20 seed plant species. Estimated divergence time confidence intervals are shown at each internal node as teal bars and the average divergence time is indicated above the bar. Calibrated nodes are indicated by red dots. The Magnoliales and Laurales shared WGD is indicated with a blue star.

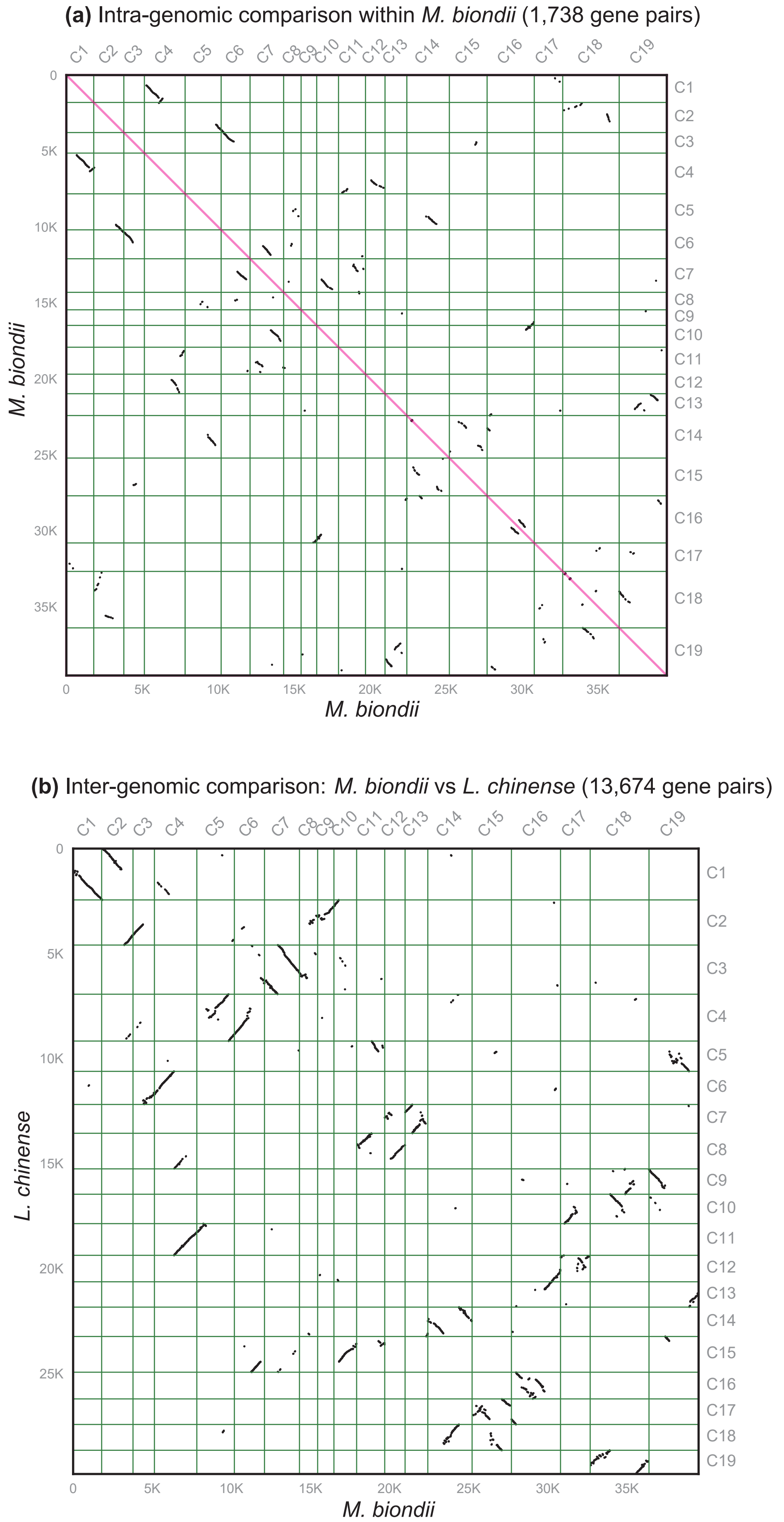

Figure S6. Intra-genomic comparison (a) within *M. biondii.* (b). of *M. biondii* and *L. chinense* genomes.
