# Supplementary material for: The genome of Magnolia biondii Pamp. provides insights into the evolution of Magnoliales and biosynthesis of terpenoids
Source: Hortic Res. 2021 Mar 1;8:38. doi: 10.1038/s41438-021-00471-9 (PMC7917104; doi:10.1038/s41438-021-00471-9)
Supplement: Supplementary file 2 — Supplementary tables [file 41438_2021_471_MOESM2_ESM.pdf]

Table S1. Sequencing statistics.

DNA-seq	Raw data					Clean data			
	Library type	Read length (max; mean)	Total number of reads	Total number of bases (Gb)	Depth (X)	Read length (bp)	Total number of reads (bp)	Total number of bases (Gb)	Depth (X)
10 x Genomics	Chromium	150	1,169,695,614	175.45	80.00	150	908,810,764	136.32	61.96
Pacific SMRT	20kb library	100,846;10324	5,953,700	66.78	30.35	NA	NA	NA	NA
HI-C		100	768,917,688	153.78	69.9				

RNA-seq	Raw data		Clean data		Sample
	Read number (bp)	Base number (bp)	Base number (bp)	Reads number (bp)	
FLOWER	51,130,512	4,601,746,080	2,898,235,224	33,897,488	flower
BUDA	51,847,396	4,666,265,640	3,154,682,214	36,896,868	pre-meiosis flower
BUDB	52,557,176	4,730,145,840	2,609,209,314	30,517,068	post-meiosis flower
LEAF	51,326,726	4,619,405,340	2,197,638,135	25,703,370	leaf
Total	206,861,810	18,617,562,900	10,859,764,887	127,014,794	/

Table S2. K-mer survey statistics and evaluation of Hi-C data

K-mer frequency analysis

Kmer	Kmer_num	Pkdepth	Genome size (Mb)	Used base (Gb)	Used read (Mb)	X
17	104,335,244,432	48	2,173.65	118.35	876.39	54.451

GCE software

Raw_peak	Now_node	Cvg	Genome size (Mb)	Heterozygous ratio (%)	Repeat ratio (%)
29	1,113,015,883	29.5297	2,236.75	0.73	61.83

Evaluation of Hi-C reads

total reads pairs	768917688
reads1 mapping rate	0.8556
reads2 mapping rate	0.8558
valid reads pairs	281232993
Pair Type %(L-I-O-R)	25% - 25% - 25% - 25%
unique reads	171064287
Hi-C contacts reads	158069682
Long Range (>20Kb) reads	24336227

Table S3. Statistics of the raw genome assemblies.

	Canu + corrected	wtdbg + raw reads	wtdbg + corrected reads	SamrtDenovo+ corrected	Flye	Miniasm
Total Contig length (Gb)	1.87	1.75	1.53	1.48	1.78	2.20
Number of contigs	16,431	14,273	12,028	14,204	14,263	15,713
Contig N50 (Kb)	185.16	265.34	253.33	147.33	275.71	267.112
Contig N90 (Kb)	47.29	60.17	62.11	47.68	60.76	59.46
Max contig length(Kb)	1,848.39	2,202.97	1,532.87	1,049.52	2,205.34	2,125.10
Complete BUSCOs (%)	61.5	32.1	60.8	4.9	72.0	75.6
Complete and single-copy BUSCOs (%)	57.7	31.8	59.5	4.6	69.3	73.5
Complete and duplicated BUSCOs (%)	3.8	0.3	1.3	0.3	2.7	2.1
Fragmented BUSCOs (%)	16.0	22.1	18.5	10.8	12.9	10.0

Table S4. Repeat annotations of the *M. biondii* genome assembly

Repeat elements	Type	% of genome	Length (bp)
Type I: Retrotransposon elements	SINE	0.01	252,416
	LINE	4.47	99,544,077
	LTR	58.06	1,291,718,910
	LTR/Copia	26.28	584,683,554
	LTR/Gypsy	29.42	654,355,377
Type II: DNA transposon	DNA	5.86	130,503,028
Type III: Tandem repeats	Satellite	0.24	5,540,573
	Simple_repeat	0.79	17,626,796
Others		0.32	7,240,517
Total repeat		66.48	1,478,819,185

Table S5. Gene annotation statistics of the *M. biondii* assembly and transcriptome assembly statistics

Protein-coding genes prediction

Species	Genome size (Mb)	Gene number	Average /median gene length (bp)	Average /median coding sequence length (bp)	Average exons per gene	Average /median exon length (bp)	Average /median intron length (bp)	C (Complete BUSCOs)	S (Complete and single-copy BUSCOs)	D (Complete and duplicated BUSCOs)	F (Fragmented BUSCOs)	M (Missing BUSCOs)
M. biondii	**2,224**	**47,547**	**10,980/3,701**	**957/675**	**4.45**	**215/133**	**2,774/525**	**1,232 (89.60%)**	**1,171 (85.16%)**	**61 (4.44%)**	**78 (5.67%)**	**65 (4.73%)**
A. trichopoda	706	31,494	12,546/7,376	1,453/1,203	6.96	209/118	1,873/486	1,351 (98.25%)	672 (48.87%)	679 (49.38%)	9 (0.65%)	15 (1.09%)
A. thaliana	119	27,628	1,868/1,554	1,217/1,041	5.11	237/134	158/98	1,370 (99.63%)	1,360 (98.91%)	10 (0.73%)	2 (0.14%)	3 (0.22%)
C. kanehirae	730	26,531	7,611/4,609	1,320/1,095	5.42	244/135	1,422/524	1,242 (90.33%)	1,160 (84.36%)	82 (5.96%)	39 (2.84%)	94 (6.84%)
L. chinense	1,742	35,269	10,589/5,648	1,266/1,077	4.89	259/141	2,396/571	1,109 (80.65%)	1,016 (73.89%)	93 (6.76%)	164 (11.93%)	102 (7.42%)
O. sativa	374	41,070	3,437/2,545	1,421/1,212	5.66	251/128	433/145	1,371 (99.71%)	1,020 (74.18%)	351 (25.53%)	0 (0%)	4 (0.29%)

Non-coding RNA genes in the genome of *M. biondii*

			rRNA					snRNA			
Type	miRNA	tRNA	rRNA	18S	28S	5.8S	5S	snRNA	CD-box	HACA-box	splicing
Copy (w)	109	904	959	243	480	98	138	3,713	3,383	67	263
Average length (bp)	124	75	292	765	134	153	104	110	106	134	153
Total length (bp)	13,493	68,172	279,809	185,909	64,541	15,019	14,340	407,934	358,695	8,973	40,266
% of genome	0.000607	0.003065	0.012578	0.008357	0.002901	0.000675	0.000645	0.018338	0.016125	0.000403	0.001810

Transcriptome assembly

	FLOWER	BUDA	BUDB	LEAF
Complete BUSCOs	719 (52.29%)	880 (64.00%)	847 (61.60%)	753 (54.76%)
Complete and Single-copy BUSCOs	563 (40.95%)	672 (48.87%)	612 (44.51%)	583 (42.40%)
Complete and Duplicated BUSCOs	156 (11.35%)	208 (15.13%)	235 (17.09%)	170 (12.36%)
Fragmented BUSCOs	313 (22.76%)	300 (21.82%)	339 (24.65%)	354 (25.75%)
Missing BUSCOs	343 (24.95%)	195 (14.18%)	189 (13.75%)	268 (19.49%)

Table S6. Functional annotation of predicted genes in *M. biondii* genome

Values	NR	Swissprot	KEGG	COG	TrEMBL	Interpro	Overall	Unannotated
Number	38,517	28,121	28,377	12,589	37,791	28,484	39,111	8,436
Percentage	81.01%	59.14%	59.68%	26.48%	79.48%	59.91%	82.26%	17.74%

Table S7. Data used for phylogenetic reconstruction of angiosperms.

Taxa	Database	Accession	Url
Amborella trichopoda	NCBI	GCF_000471905.2	https://www.ncbi.nlm.nih.gov/assembly/GCF_000471905.2/
Arabidopsis thaliana	TAIR10	NA	https://www.arabidopsis.org/download/index-auto.jsp?dir=%2Fdownload_files%2FGenes%2FTAIR10_genome_release
Cinnamomum kanehirae	NCBI	GCA_003546025.1	https://www.ncbi.nlm.nih.gov/genome/57158?genome_assembly_id=405569
Liriodendron chinense	Hardwood Genomics Project	NA	https://www.hardwoodgenomics.org/Genome-assembly/2630420?tripal_pane=group_downloads
Oryza sativa	NCBI	GCF_001433935.1	https://www.ncbi.nlm.nih.gov/assembly/GCF_001433935.1/
Picea abies	Picea abies v1.0	NA	ftp://plantgenie.org/Data/ConGenIE/
Sorghum bicolor	NCBI	GCF 000003195.3	https://www.ncbi.nlm.nih.gov/assembly/GCF_000003195.3
Vitis vinifera	12X v0	NA	http://www.genoscope.cns.fr/externe/Download/Projets/Projet_ML/data/12X/
Annona muricata	OneKp	NA	ftp://parrot.genomics.cn/gigadb/pub/10.5524/100001_101000/100627/assemblies/
Aristolochia elegans	OneKp	NA	ftp://parrot.genomics.cn/gigadb/pub/10.5524/100001_101000/100627/assemblies/
Ascarina rubricaulis	OneKp	NA	ftp://parrot.genomics.cn/gigadb/pub/10.5524/100001_101000/100627/assemblies/
Canella winterana	OneKp	NA	ftp://parrot.genomics.cn/gigadb/pub/10.5524/100001_101000/100627/assemblies/
Illicium floridanum	OneKp	NA	ftp://parrot.genomics.cn/gigadb/pub/10.5524/100001_101000/100627/assemblies/
Illicium parviflorum	OneKp	NA	ftp://parrot.genomics.cn/gigadb/pub/10.5524/100001_101000/100627/assemblies/
Laurelia sempervirens	OneKp	NA	ftp://parrot.genomics.cn/gigadb/pub/10.5524/100001_101000/100627/assemblies/
Magnolia grandiflora	OneKp	NA	ftp://parrot.genomics.cn/gigadb/pub/10.5524/100001_101000/100627/assemblies/
Nymphaea sp.	OneKp	NA	ftp://parrot.genomics.cn/gigadb/pub/10.5524/100001_101000/100627/assemblies/
Sarcandra glabra	OneKp	NA	ftp://parrot.genomics.cn/gigadb/pub/10.5524/100001_101000/100627/assemblies/
Saruma henryi	OneKp	NA	ftp://parrot.genomics.cn/gigadb/pub/10.5524/100001_101000/100627/assemblies/

Table S8. KEGG enrichment of *M. biondii* unique gene families.

Pathway ID	Pathway	Gene number	Total gene	Pvalue	negLog10_Pvalue
ko03040	Spliceosome	650	1342	5.46E-174	173.262638
ko03030	DNA replication	607	1213	6.65E-171	170.176915
ko04120	Ubiquitin mediated proteolysis	429	1005	3.71E-90	89.4303038
ko03018	RNA degradation	375	842	5.47E-85	84.2616164
ko00460	Cyanoamino acid metabolism	264	484	1.15E-83	82.9387606
ko00940	Phenylpropanoid biosynthesis	284	807	8.65E-40	39.062995
ko03013	RNA transport	276	841	1.08E-32	31.9654217
ko00500	Starch and sucrose metabolism	313	1025	1.54E-30	29.8114315
ko04626	Plant-pathogen interaction	476	1808	8.78E-29	28.056534
ko00960	Tropane, piperidine and pyridine alkaloid biosynthesis	69	131	1.37E-21	20.8624671
ko00130	Ubiquinone and other terpenoid-quinone biosynthesis	67	150	3.46E-16	15.4611473
ko00950	Isoquinoline alkaloid biosynthesis	71	164	3.50E-16	15.4556506
ko00400	Phenylalanine, tyrosine and tryptophan biosynthesis	77	186	4.24E-16	15.3730527
ko01110	Biosynthesis of secondary metabolites	584	2720	1.90E-13	12.7211474
ko00350	Tyrosine metabolism	73	201	5.79E-12	11.2370309
ko00591	Linoleic acid metabolism	60	164	3.05E-10	9.51519824
ko00270	Cysteine and methionine metabolism	77	242	2.10E-09	8.67714111
ko00062	Fatty acid elongation	39	105	2.32E-07	6.63506947
ko00902	Monoterpenoid biosynthesis	28	65	3.39E-07	6.46973651
ko03410	Base excision repair	70	238	3.46E-07	6.46103387
ko00730	Thiamine metabolism	20	41	1.48E-06	5.82860941
ko04144	Endocytosis	109	445	6.47E-06	5.18903598
ko00360	Phenylalanine metabolism	75	289	2.19E-05	4.66045595
ko03420	Nucleotide excision repair	54	193	3.38E-05	4.47093132
ko00640	Propanoate metabolism	50	199	0.001027254	2.98832216
ko00280	Valine, leucine and isoleucine degradation	54	235	0.005338885	2.27254943
ko00410	beta-Alanine metabolism	44	192	0.01155411	1.9372635
ko03440	Homologous recombination	27	107	0.01251281	1.90264515

Table S9. GO enrichment of *M. biondii* unique gene families.

GO ID	GO Term	GO Class	Pvalue	Adjusted Pvalue	Gene number	Total gene
GO:0003676	nucleic acid binding	MF	4.27E-298	3.1436E-295	982	3397
GO:0004523	ribonuclease H activity	MF	5.43E-180	2.00E-177	278	584
GO:0004521	endoribonuclease activity	MF	1.22E-168	1.80E-166	280	616
GO:0004540	ribonuclease activity	MF	1.28E-167	1.58E-165	281	622
GO:0004518	nuclease activity	MF	2.50E-147	2.30E-145	285	689
GO:0097159	organic cyclic compound binding	MF	3.33E-112	2.45E-110	1205	6768
GO:1901363	heterocyclic compound binding	MF	3.33E-112	2.45E-110	1205	6768
GO:0016788	hydrolase activity, acting on ester bonds	MF	3.51E-95	2.15E-93	298	941
GO:0005488	binding	MF	6.62E-53	3.49E-51	1491	10651
GO:0008725	DNA-3-methyladenine glycosylase activity	MF	2.45E-13	4.30E-12	20	29
GO:0016787	hydrolase activity	MF	7.97E-11	7.08E-10	403	2756
GO:0046983	protein dimerization activity	MF	2.58E-05	1.21E-04	67	374
GO:0003677	DNA binding	MF	0.0003381	0.001456997	165	1156
GO:0015930	glutamate synthase activity	MF	0.0024541	0.009227846	6	14
GO:0016742	hydroxymethyl-, formyl- and related transferase activity	MF	0.0037113	0.013540776	6	15
GO:0045735	nutrient reservoir activity	MF	0.0051022	0.017821575	17	79
GO:0016838	carbon-oxygen lyase activity, acting on phosphates	MF	0.0062046	0.02136816	21	106
GO:0004602	glutathione peroxidase activity	MF	0.0066197	0.022482466	5	12
GO:0010333	terpene synthase activity	MF	0.0089862	0.029698702	20	102
GO:0003871	5-methyltetrahydropteroyltriglutamate-homocysteine S-methyltransferase activity	MF	0.0138667	0.042405784	5	14
GO:0005337	nucleoside transmembrane transporter activity	MF	0.0138667	0.042405784	5	14
GO:0006260	DNA replication	BP	2.49E-11	2.55E-10	30	89
GO:0006284	base-excision repair	BP	1.62E-10	1.37E-09	20	37
GO:0006259	DNA metabolic process	BP	7.26E-08	4.42E-07	61	290
GO:0042147	retrograde transport, endosome to Golgi	BP	0.0013993	0.005544725	5	9
GO:0006904	vesicle docking involved in exocytosis	BP	0.0020849	0.007961451	7	18
GO:0006281	DNA repair	BP	0.0025014	0.009358175	25	126
GO:1901642	nucleoside transmembrane transport	BP	1.39E-02	4.24E-02	5	14
GO:0000786	nucleosome	CC	7.25E-08	4.42E-07	26	88
GO:0044427	chromosomal part	CC	9.44E-08	5.66E-07	32	120
GO:0005694	chromosome	CC	6.028E-06	3.12839E-05	33	142
GO:0005664	nuclear origin of replication recognition complex	CC	0.0005061	0.002156142	6	11
GO:0030906	retromer complex, inner shell	CC	0.0013993	0.005544725	5	9

Table S10. KEGG enrichment of the *M. biondii* expanded gene families.

Pathway ID	Pathway	Gene number	Total gene	Pvalue
ko02010	ABC transporters	209	286	4.04E-31
ko00052	Galactose metabolism	191	277	1.60E-23
ko00945	Stilbenoid, diarylheptanoid and gingerol biosynthesis	99	118	2.55E-23
ko00941	Flavonoid biosynthesis	135	193	6.91E-18
ko00604	Glycosphingolipid biosynthesis - ganglio series	64	74	6.32E-17
ko00531	Glycosaminoglycan degradation	78	98	4.58E-16
ko04710	Circadian rhythm - mammal	60	78	1.61E-11
ko00600	Sphingolipid metabolism	79	116	3.58E-10
ko03020	RNA polymerase	118	194	1.22E-09
ko03008	Ribosome biogenesis in eukaryotes	188	344	6.31E-09
ko00561	Glycerolipid metabolism	117	198	1.55E-08
ko00511	Other glycan degradation	89	142	1.67E-08
ko00510	N-Glycan biosynthesis	96	161	1.60E-07
ko00904	Diterpenoid biosynthesis	48	68	1.84E-07
ko00240	Pyrimidine metabolism	212	421	2.89E-06
ko00040	Pentose and glucuronate interconversions	131	244	4.03E-06
ko03050	Proteasome	115	213	1.08E-05
ko04075	Plant hormone signal transduction	349	742	1.12E-05
ko00402	Benzoxazinoid biosynthesis	16	18	2.03E-05
ko03010	Ribosome	363	779	2.08E-05
ko00906	Carotenoid biosynthesis	67	114	2.19E-05
ko00944	Flavone and flavonol biosynthesis	39	60	5.31E-05
ko00053	Ascorbate and aldarate metabolism	49	81	9.83E-05
ko01100	Metabolic pathways	2017	4848	0.0003098
ko00603	Glycosphingolipid biosynthesis - globo series	21	29	0.000321
ko00592	alpha-Linolenic acid metabolism	65	119	0.0005383
ko00190	Oxidative phosphorylation	141	288	0.0005881
ko00900	Terpenoid backbone biosynthesis	61	112	0.0008655
ko00195	Photosynthesis	74	144	0.0022872
ko00902	Monoterpenoid biosynthesis	37	65	0.0031434
ko00901	Indole alkaloid biosynthesis	12	16	0.0042181
ko01040	Biosynthesis of unsaturated fatty acids	45	86	0.0101142
ko00450	Selenocompound metabolism	42	80	0.0118102
ko03015	mRNA surveillance pathway	161	361	0.0244063
ko00480	Glutathione metabolism	82	175	0.0264966
ko00966	Glucosinolate biosynthesis	14	23	0.0304717
ko00520	Amino sugar and nucleotide sugar metabolism	98	215	0.037331
ko00908	Zeatin biosynthesis	41	83	0.0407805
ko00073	Cutin, suberine and wax biosynthesis	39	79	0.0456582

Table S11. GO (level 3) enrichment of the *M. biondii* expanded gene families.

GO Term	GO Class	Pvalue	Qvalue	Adjusted Pvalue	Gene number	Total gene
oxidoreductase activity	MF	5.25E-39	36.11	7.77E-37	908	1521
transferase activity	MF	1.02E-17	15.42	3.78E-16	1435	2805
peroxidase activity	MF	2.00E-15	13.23	5.93E-14	107	138
transmembrane transporter activity	MF	9.37E-15	12.64	2.31E-13	402	691
lyase activity	MF	9.39E-14	11.81	1.55E-12	174	261
ion binding	MF	2.47E-12	10.52	3.05E-11	2402	5010
drug transporter activity	MF	6.79E-11	9.14	7.18E-10	55	65
substrate-specific transporter activity	MF	8.10E-08	6.30	5.00E-07	177	299
carbohydrate binding	MF	9.47E-06	4.30	5.01E-05	94	152
electron transporter, transferring electrons within the cyclic electron transport pathway of photosynthesis activity	MF	0.000257	2.92	0.001189	10	10
enzyme inhibitor activity	MF	0.000338	2.83	0.001471	62	100
carbohydrate derivative binding	MF	0.002346	2.04	0.009136	16	20
signaling receptor activity	MF	0.002836	1.97	0.010762	35	54
signal transducer activity	MF	0.004577	1.78	0.016522	46	76
response to chemical stimulus	BP	1.85E-24	21.86	1.37E-22	145	177
single-organism metabolic process	BP	6.46E-19	16.50	3.18E-17	1117	2116
single-organism transport	BP	4.16E-14	12.06	8.79E-13	638	1173
response to endogenous stimulus	BP	9.44E-14	11.81	1.55E-12	78	95
single-multicellular organism process	BP	2.30E-10	8.65	2.23E-09	78	104
establishment of localization	BP	2.41E-10	8.65	2.23E-09	728	1404
reproductive process	BP	3.77E-09	7.48	3.29E-08	64	84
multi-organism reproductive process	BP	6.59E-09	7.29	5.13E-08	60	78
multi-multicellular organism process	BP	6.59E-09	7.29	5.13E-08	60	78
cell wall organization or biogenesis	BP	1.77E-08	6.88	1.31E-07	79	112
cellular process involved in reproduction	BP	3.19E-08	6.67	2.15E-07	60	80
single-organism cellular process	BP	5.53E-07	5.50	3.15E-06	1033	2114
response to biotic stimulus	BP	5.75E-06	4.50	3.15E-05	24	27
cellular component organization	BP	0.001065	2.36	0.00438	178	338
multicellular organismal development	BP	0.003972	1.83	0.014695	18	24
response to other organism	BP	0.013005	1.36	0.043744	8	9
primary metabolic process	BP	0.014508	1.33	0.047271	2468	5467
intrinsic to membrane	CC	2.97E-12	10.47	3.38E-11	474	856
cell periphery	CC	2.15E-07	5.90	1.27E-06	104	162
external encapsulating structure	CC	1.31E-05	4.17	6.69E-05	62	93
membrane part	CC	9.69E-05	3.33	0.000463	519	1046
